# Optometrist-guided versus self-driven subjective refraction using tunable optics: quantifying the professional’s impact

**DOI:** 10.1016/j.optom.2026.100611

**Published:** 2026-03-01

**Authors:** Raquel Salvador-Roger, Abinaya Priya Venkataraman, Vicente Micó, Alberto Domínguez-Vicent, José J. Esteve-Taboada

**Affiliations:** a Department of Optics and Optometry and Vision Sciences. University of Valencia. 46100 Burjassot, Spain; b Unit of Optometry, Division of Eye and Vision, Department of Clinical Neuroscience, Karolinska Institute, 171 77 Stockholm, Sweden

**Keywords:** Self-refraction, Refractive error, Tunable lens, Subjective refraction

## Abstract

**Purpose:**

To evaluate the influence of professional guidance on a novel subjective refraction method combining a tunable liquid lens (TLL) and a Stokes lens, and to determine whether clinician involvement affects refractive accuracy, visual outcomes, or testing time compared with a participant-driven approach.

**Methods:**

Sixty-six participants (18–44 years old) underwent monocular subjective refraction in one randomly selected eye under two conditions: a professional-guided refraction (ORx) performed by an experienced optometrist, and a do-it-yourself (DIY) condition where participants completed the procedure independently. Both methods used the same optical system for continuous spherical and astigmatic adjustment. Three consecutive measurements were taken under repeatability conditions. Refractive components (M, J_0_, J_45_), visual acuity, and measurement time were compared using Passing–Bablok regression and non-parametric tests.

**Results:**

A strong agreement was found between both methods, with slopes including the value 1 within the 95% confidence intervals. Intercepts for the spherical equivalent (M) and time did not include zero, indicating a small systematic bias: M values were slightly more positive and measurement time longer in the ORx condition. This effect was more evident in hyperopic and near-emmetropic eyes. No statistically significant differences were observed between methods (p > 0.05). Both techniques showed a learning effect with reduced time across repetitions.

**Conclusion:**

DIY subjective refraction achieved comparable accuracy and visual outcomes to professional-guided procedures. While clinician supervision improved consistency and accommodative control, the DIY approach demonstrated feasibility as a complementary and accessible option for hybrid or remote refraction applications.

## Introduction

The precise evaluation of refractive errors is a fundamental aspect of optometry and ophthalmology.^1^ Although objective techniques have evolved considerably, subjective refraction remains the reference standard for determining the final prescription, as it takes into account individual perceptual preferences and provides the most accurate correction for optimal visual performance. The interactive nature of subjective refraction, in which the patient's responses guide the refinement of the prescription, ensures a level of personalization that objective methods cannot yet achieve.^2^

Efforts to continuously improve the efficiency and accuracy of subjective refraction have led to the development of new optical systems capable of continuous and dynamic power adjustment.^3^, ^4^, ^5^, ^6^, ^7^ Tunable liquid lenses (TLLs) and adjustable astigmatic elements, such as Stokes lenses, are among the most promising technologies in this field.^8^^,^^9^ These devices allow for seamless spherical and cylindrical power variation within compact, lightweight setups and align well with the vector representation of refractive errors.^10^, ^11^, ^12^, ^13^, ^14^

In a previous study, we explored a DIY subjective refraction approach that allowed participants to perform their own refraction using a tunable lens system. This study demonstrated the feasibility and potential accuracy of this patient-driven method, highlighting its potential as a simplified, accessible approach to refractive evaluation.^15^

However, it remains unclear how the absence of professional guidance might affect the final results. During a traditional subjective refraction, the optometrist plays a key role in interpreting patient feedback, determining when to conclude the refinement process, and guaranteeing the stability and reliability of the measurements. These aspects could lead to differences in accuracy, consistency, and participant confidence compared to a completely self-directed procedure.^16^

The present study builds upon previous findings by directly comparing the DIY subjective refraction method with a professionally guided approach, both of which use the same optical system. This study addresses whether patient-driven refraction can achieve comparable precision when performed independently or if professional involvement is essential to maintain clinical reliability.

## Methods

### Participants

A total of 66 subjects aged between 18 to 44 years were included in the present study. Inclusion criteria were no history of known ocular disease, and no previous ocular surgery.

The study was approved by the Swedish Ethical Review Authority, and the study procedures adhered to the tenets of the Declaration of Helsinki. Each participant signed an informed consent form after receiving a detailed explanation of the purpose, nature, and possible consequences of the study.

### Instrumentation

This study used the same method to assess monocular subjective refraction in one eye of each subject but performed in two different ways: one conducted by a well-trained eye-care practitioner (ORx) and a second one where the main process relied on participants (DIY). Illumination was checked close to subjects’ eye level, and the same illumination level was maintained for all measurements. For the measurements, an optotype was placed at a distance of 6 meters. A Topcon CC-100 Early Treatment of Diabetic Retinopathy Study (ETDRS) chart in logMAR format was used, displaying lines of five letters arranged in random order. The measurement techniques and the eye measured were randomized. Three consecutive measurements were taken with tunable lens and guided method under repeatability conditions.^17^^,^^18^ The subjective refraction values, visual acuity and time required were recorded.

### Refractive examination procedure

#### Professionally guided approach (ORx)

Subjective refraction was performed using a system comprising a liquid lens for spherical adjustments^19^ and a Stokes lens for astigmatic correction.^9^ This Stokes lens consisted of two cylindrical lenses of the same power but opposite sign (±1.75 D), which could be rotated in opposite directions to achieve astigmatic powers ranging from 0 D to 3.50 D. This method allowed direct measurement of the refractive error components in rectangular notation (M, J_0_, J_45_).

1. Positioning the Circle of Least Confusion (COLC):
  The liquid lens was adjusted to correct the spherical equivalent (M) by bringing the COLC to the retinal plane. An initial spherical estimate, derived from objective refraction, was used as the starting point. Positive defocus was introduced to control accommodation, and spherical power was gradually reduced in 0.25 D steps while monitoring VA. The end point was considered reached when fewer than three letters of the next optotype line could be correctly identified, and the immediately preceding lens position was accepted as the maximum positive lens providing best visual acuity. The resultant spherical value represented the M component of the refraction.
2. Astigmatic Component Measurement (J_0_ and J_45_):
  A Stokes lens was mounted behind the tunable lens to measure the orthogonal components of astigmatism.
3. Determination of J_0_ and J_45_:
  The Stokes lens was oriented with its axes aligned at 0° and 90° for determining J_0_. For J_45_ determination, the same Stokes lens was oriented this time with its axes at 45° and 135°. J_0_ and J_45_ might not be measured simultaneously since only one Stokes lens was used. To adjust the astigmatic power, the lens was rotated incrementally to optimize visual clarity. The subject was asked to report when the image did not improve anymore. This process was repeated twice, one to achieve J_0_ and another to achieve J_45_.
4. Visual Acuity Assessment:
  The final combination of spherical and astigmatic corrections (M, J_0_, J_45_) was converted to polar notation and the cylindrical component was introduced into the Stokes lens for VA measurement. LogMAR VA was recorded.

#### Do-it-yourself refraction (DIY)

In the do-it-yourself method, the steps followed coincide with those of the refraction using a liquid lens and Stokes lens. Nevertheless, in this case, the subject actively participated in adjusting the optical elements of the refraction system, specifically the liquid and Stokes lenses, under the optometrist's surveillance. This method was designed to empower subjects to identify the refractive settings that provided optimal visual acuity while maintaining a structured and reliable process.^15^

### Statistical analysis

Only data from one eye of each participant were measured to avoid the possibility of artificially reducing the confidence interval around the limits of agreement.^20^ Descriptive statistics were used to summarize the basic demographics of the results obtained by each method. The normality of the distribution was evaluated with the Kolmogorov-Smirnov test.

The coefficient of repeatability (CoR) was used to assess repeatability and was computed as 1.96 × $S_{w}$, where the within-subject standard deviation was obtained from a one-way ANOVA with subject included as a factor.^20^

Since the data did not follow a normal distribution, the agreement between the techniques was evaluated by the Passing-Bablok regression test.^21^^,^^22^ For the proper application of this method, the two requirements regarding continuous data distribution and linear relationship between both data sets were checked. The cumulative sum linearity test (cusum test) was used to confirm whether residuals are randomly distributed above and below the regression line. A p-value less than 0.05 was considered statistically significant, which denotes nonlinearity between both data sets. Scatter plots are shown with the following parameters represented: regression line with its equation (y = ax + b), identity line (y = x) and 95% confidence intervals for the intercept and the slope of the regression line.

## Results

**Fig. 1:**
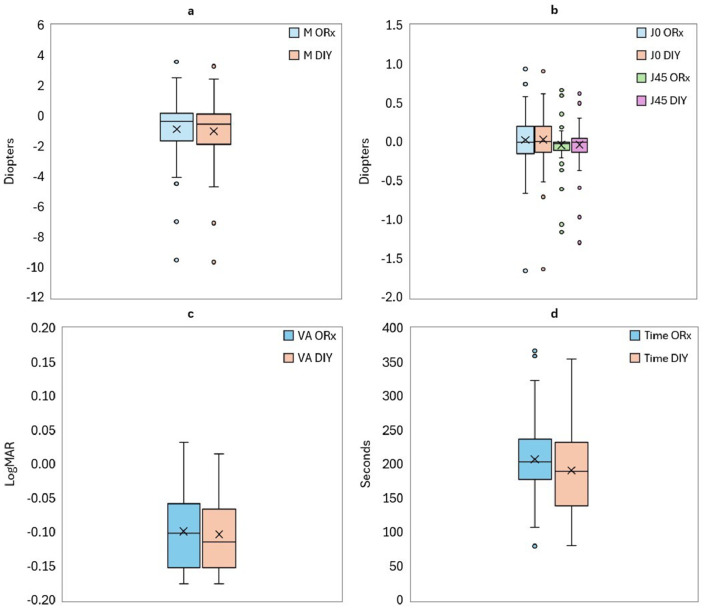
Boxplots showing the distribution of spherical equivalent component (a), astigmatic components (b), visual acuity (c), and measurement time (d) obtained with the optometrist-guided (ORx) and do-it-yourself (DIY) subjective refraction methods. Central lines represent medians; boxes indicate interquartile ranges; whiskers denote data range.

The mean age of the participants was 29 ± 9 years, and the average spherical equivalent was -0.98 ± 2.28 D. Fig. 1 shows the mean values for M (panel a), J_0_ and J_45_ (panel b), best VA (panel c), and time taken (panel d) for each method (ORx and DIY). The median values for both methods are similar across all panels, with comparable interquartile ranges (IQR) in panels a and b. However, in panels c and d, the IQR varies between methods, particularly in visual acuity (panel c) and time taken (panel d), indicating greater variability in these measurements for the DIY method.

The repeatability coefficient for the M component was ±0.33 D. For the astigmatic components, the CoR reached ±0.19 D for J_0_ and ±0.17 D for J_45_. Visual acuity showed a CoR of ±0.05 logMAR, while the coefficient associated with measurement duration was ±72 s.

**Fig. 2:**
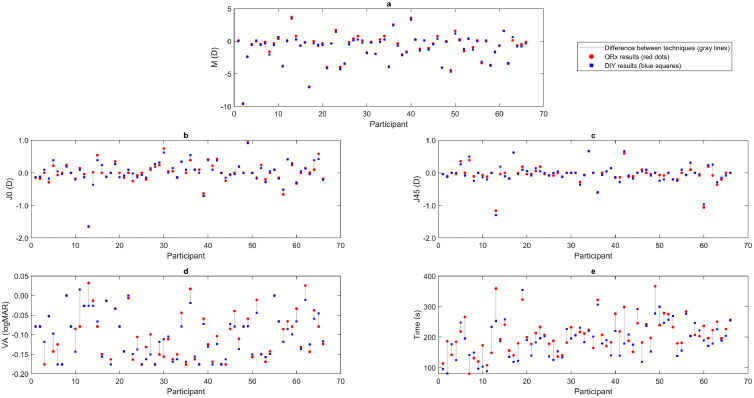
Scatter plot showing the mean value of the repeated measurements obtained for each participant with the optometrist-guided (ORx, red dots) and do-it-yourself (DIY, blue squares) methods. Each pair of symbols represents the average data from one subject under both conditions.

Values obtained for each technique and each participant are represented in Fig. 2. The scatter plots display the results for each participant, with red dots representing ORx values, blue squares showing DIY values, and grey lines highlighting the differences between the two methods. For M, the values ranged from -9.52 to 3.72 D with the ORx method and from -9.66 to 3.44 D with the DIY method. The maximum and minimum differences found in the M component are 0.52 and 0 D, respectively, where 73% of the cases show less than 0.25 D difference, and 39% are less than 0.10 D. For J_0_, the ranges were -1.66 to 0.94 D (ORx method) and -1.64 to 0.91 D (DIY method). Similarly, values ranged from -1.16 to 0.67 D (ORx method) and from -1.30 to 0.67 D (DIY method) for the J_45_ method. The maximum differences found for the astigmatic components are 0.38 and 0.33 D for J_0_ and J_45_, respectively, while the minimum for both is 0 D. 79% of the J_0_ differences and 76 % of the J_45_ differences are under the value of 0.05 D.

Given that refraction is expressed in vector form, the overall difference between methods can also be summarized by the modulus of the power vector, commonly referred to as blur strength or ΔRx,^23^^,^^24^ calculated as the square root of the sum of squares of the three refractive components. Using this metric, the mean difference in blur strength was 0.11 ± 0.20 D, with a median value of 0.08 D.

Visual acuity ranged from -0.18 to 0.03 logMAR with the ORx method and from -0.18 to 0.02 logMAR with the DIY method. The maximum and minimum differences found in VA were 0.06 and -0.09 logMAR, where only one case had a difference over 0.06 logMAR. Finally, measurement time ranged from 80 to 366 seconds for the ORx method and from 81 to 354 seconds for the DIY method, with a maximum difference between techniques of 121 seconds and a minimum of 0 seconds. In 45% of cases, differences were less than 10 seconds.

**Fig. 3:**
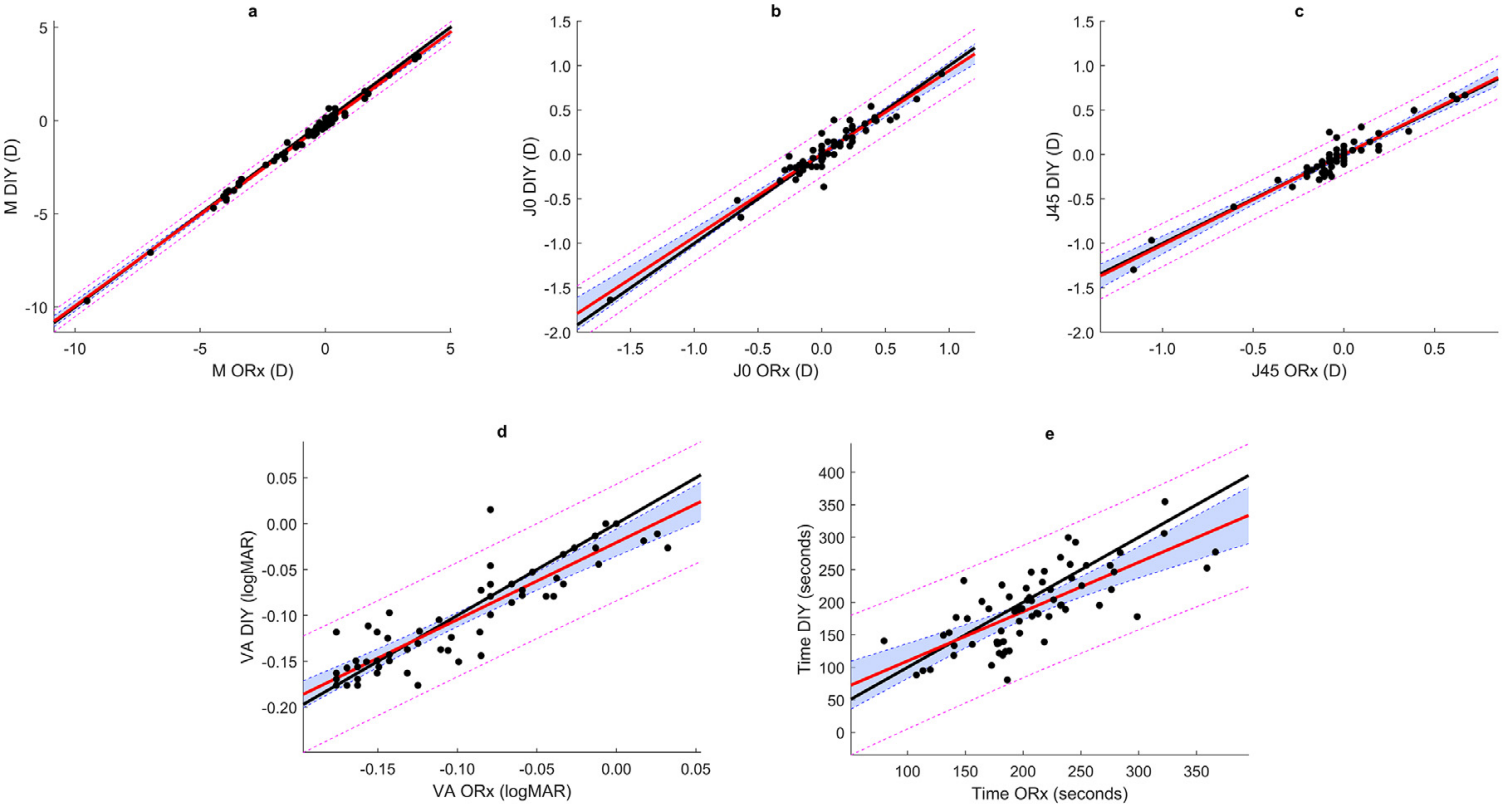
Passing–Bablok regression plots for M (a), J_0_ (b), J_45_ (c), VA (d), and time (e). The regression line is red, and the identity line is black. The colored area represents the 95% confidence intervals for the regression coefficients, and the pink dotted lines represent the 95% confidence limits.

Table 1 summarizes the linear polynomial adjustment coefficients obtained for each parameter. The largest CI for the slope was shown for the required time (0.35), whereas the M components showed the shortest interval (0.04). The CIs for intercept values only exceeded 0.05 D M component among refractive components and were set to 0.03 logMAR and 76” for the VA agreement and required time, respectively. In all cases, no significant differences were found at the statistical level between the techniques (p > 0.1). Fig. 3 shows the Passing-Bablok regression test for the M (panel a), J_0_ (panel b), J_45_ (panel c), VA (panel d) and time (panel e). As it can be seen, the identity line is included in the CIs for the astigmatic components. For the M, VA and time taken, the identity line extends beyond the CI in some areas.

**Table 1:** Slope and intercept with their confidence intervals (CI) values obtained by adjusting to a regression line the same component measured with the different techniques (the optometrist approach (ORx) and the do-it-yourself (DIY) method).

	Slope [CI]	Intercept [CI]	p-value
M	0.98 [0.96 to 1.00]	-0.15 [-0.20 to -0.09] D	> 0.1
J_0_	0.94 [0.86 to 1.01]	0.01 [-0.02 to 0.03] D	> 0.1
J_45_	1.02 [0.94 to 1.10]	0.00 [-0.02 to 0.02] D	> 0.1
VA	0.84 [0.74 to 1.00]	-0.02 [-0.03 to 0.00] logMAR	> 0.1
Time	0.76 [0.58 to 0.93]	34 [-4 to 72] seconds	> 0.1

**Fig. 4:**
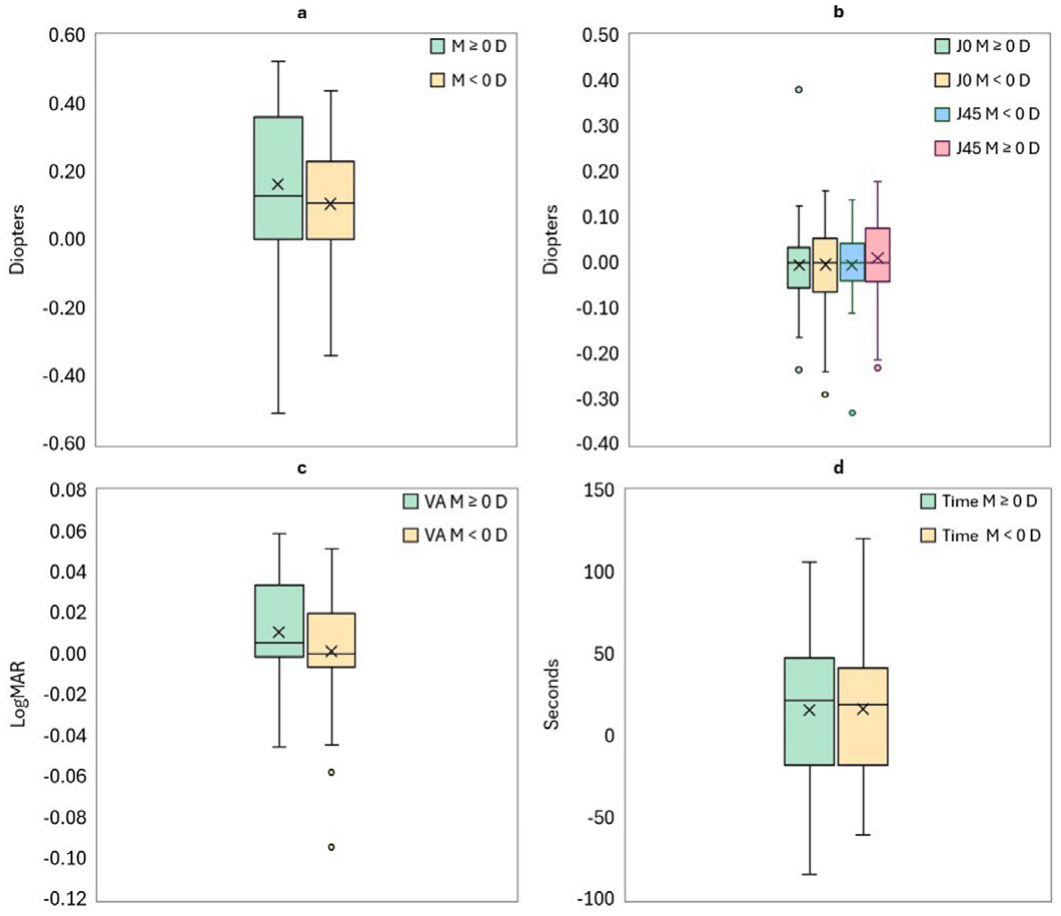
Boxplots showing the distribution of the differences between the optometrist-guided (ORx) and do-it-yourself (DIY) subjective refraction methods of spherical equivalent component (a), astigmatic components (b), visual acuity (c), and measurement time (d) obtained for participants with negative spherical equivalent (M < 0 D) and positive or zero spherical equivalent (M ≥ 0 D).

Fig. 4 shows the boxplots of the differences between methods according to the spherical equivalent of the participant. Spherical equivalent is divided between negative M values and M values positives or equal to zero diopters. In Fig. 4a, the median value difference for the M component for positive refractive errors (M ≥ 0 D) was 0.13 D [IQR: 0 – 0.36 D]. For negative refractive errors (M < 0 D), the median value difference was 0.11 D [IQR: 0 – 0.23 D]. Regarding the astigmatic components (Fig. 4b), the median values of the differences for positive refractive errors were 0.00 D [IQR: -0.05 – 0.04 D] and 0.00 D [IQR: -0.04 – 0.07 D] for J_0_ and J_45_, respectively. For negative refractive errors, the median difference was 0.00 D [IQR: -0.06 – 0.05] for J_0_ and 0.00 D [IQR: -0.04 – 0.04 D] for J_45_. In Fig. 4c, the median values of the differences for the achieved VA were 0.01 logMAR [IQR: -0.00 – 0.03 logMAR] and 0.00 logMAR [IQR: -0.01 – 0.02 logMAR] for positive and negative refractive errors, respectively. Concerning the required time (Fig. 4d), the median value for the differences were 22 seconds [IQR: -18 – 47seconds] and 19 seconds [IQR: -18 – 42 seconds] for positive and negative refractive errors, respectively.

## Discussion

The present study aimed to evaluate the role of the optometrist during the performance of a novel subjective refraction routine based on a tunable liquid lens (TLL) and a Stokes lens. The comparison between the optometrist-guided refraction (ORx) and the do-it-yourself (DIY) conditions allowed us to assess whether the clinician’s involvement significantly influences the outcomes of this simplified approach.

Regarding repeatability, the M component exhibited the highest CoR, while the astigmatic components presented CoR values below ±0.20 D. These outcomes are in line with those previously reported for the DIY method,^15^ which showed CoRs of ±0.38 D for M and ±0.21 D for both J_0_ and J_45_. Although marginally lower CoR values were observed with ORx, the overall magnitude of the differences between methods was small and remained within clinically acceptable limits.

A comparable trend was found for visual acuity, with CoR values of ±0.06 logMAR for the DIY method and ±0.05 logMAR for ORx. In contrast, repeatability in measurement time differed more noticeably, with a CoR of ±55 s for the DIY approach and ±72 s for ORx, indicating greater between-measurement variability in the ORx method.

Overall, the results showed a high level of agreement between both techniques, with no statistically significant differences in any of the measured components. The slopes of the Passing–Bablok regressions consistently included the value 1 within their confidence intervals except for the time required, confirming proportional agreement between methods for the other variables. However, for both the M component and the required time, the intercept values did not include zero as can be seen in Table 1. The non-zero intercept observed for the M component (-0.15 D) denotes a systematic shift between methods rather than a random discrepancy. In practical terms, this reflects a slightly more positive (less myopic) outcome for the professional-guided refraction, consistent with better accommodative relaxation under clinician supervision. Although the difference is small and within clinically acceptable limits, it reinforces the value of professional control in guiding subjective decisions near the refraction endpoint.^25^ Both astigmatic components (J_0_ and J_45_) exhibited excellent agreement between methods, with slopes and intercepts near unity and zero, respectively. These results suggest that the participants were able to reliably adjust the Stokes lens orientation and magnitude, even without professional supervision, highlighting the intuitive nature of the tunable system for astigmatic refinement. The strong agreement in visual acuity outcomes further supports the validity of the DIY approach. The small deviation of the slope (0.84) indicates slightly lower VA values for the DIY condition, which may be attributed to subtle undercorrection or less precise endpoint perception by the participant. Nonetheless, these differences remained well below thresholds of clinical significance, confirming the robustness of the system for visual optimization.

Interestingly, time was the only parameter for which the slope did not include the value 1 within its confidence interval. The slope below unity (0.76) and positive intercept (34”) indicate that while the DIY approach generally required less time for longer refractions, shorter measurements tended to take slightly longer compared to the professional-guided condition. This pattern suggests that participants became more efficient as they gained familiarity with the procedure, whereas the clinician maintained a more constant pace across cases. Such a trend aligns with the notion of a learning effect intrinsic to self-driven methods.

In order to evaluate whether there was a learning effect or not, some checking was made for M and the time required. Median differences for M were 0.37 D [IQR: 0 – 0.39 D] and 0.20 D [IQR: 0 – 0.39 D] for the first and the third measurement. Regarding time, median differences were 20 seconds [IQR: -173 – -34 seconds] and 20 second [IQR: -65 – -7 seconds] for the first and the third measurement. Although the inter-method differences in task duration did not reach statistical significance (Wilcoxon signed-rank test, p-value M = 0.55; p-value time = 0.88), the interquartile range for the time markedly decreased from 90 seconds in the first repetition to 44 seconds in the third, indicating greater consistency between methods after repeated testing. This reduction in variability suggests a potential learning or adaptation effect, even in the absence of statistically significant mean changes.

In general, the DIY condition resulted in shorter measurement times. This is likely due to the absence of verbal feedback and decision-making exchanges between the participant and the clinician, which naturally reduces the total duration. Despite these time differences (which decrease after repeated testing), overall accuracy was comparable between the two procedures, suggesting that the DIY approach could be a valid alternative when professional supervision is not available. In terms of time, other subjective refraction approaches such as new methodologies or hybrid technologies combining objective and subjective refraction had reduced the required time to perform the measurements.^4^^,^^26^, ^27^, ^28^

Moreover, when the value of the spherical equivalent was considered, this trend was more pronounced in hyperopic and near-emmetropic participants, whose M values under professional guidance tended to be even more positive than those obtained in the DIY condition as can be seen in Fig. 4a. This pattern suggests that optometrist supervision can help control accommodation and guide the endpoint, particularly in participants prone to overaccommodation. Similarly, Rodríguez-López et al.^28^ found a standard deviation up to ±0.65 D when the blur-detection task was performed in an unsupervised way. From a clinical perspective, the median differences observed (0.12 D) are small compared with the variability observed between independent optometrists measuring the same population,^25^^,^^26^ indicating that the practical impact of professional guidance in this context is limited. Nevertheless, even such minimal systematic biases could have perceptual implications for certain individuals, especially hyperopic participants or those with limited accommodative flexibility. In contrast, no relevant differences were observed in the astigmatic components (J_0_ and J_45_) or in visual acuity, which supports the robustness of the DIY measurements for these parameters.

The narrower interquartile ranges observed in the ORx condition for measurement time further underline the standardizing role of the practitioner. The clinician likely provides a more consistent pacing and decision structure during the procedure, leading to reduced variability between individuals. However, both methods showed an evident learning effect, with shorter measurement times across consecutive repetitions.^15^ This trend suggests that familiarity with the system and procedure contributes to improved efficiency, regardless of whether the refraction is performed independently or under supervision.

From a practical standpoint, these results suggest that DIY-based subjective refraction could serve as a preliminary or screening tool, reducing examination time and professional workload, while professional-guided procedures remain preferable for final prescriptions or for patients with complex refractive profiles. The comparable refractive outcomes between the two approaches highlight the potential of self-directed refraction systems to increase accessibility in vision testing, particularly in tele-optometry or low-resource contexts.

Several limitations should be acknowledged. First, the level of accommodation could not be fully controlled and may have varied among participants, especially in the DIY condition. Second, the participants’ understanding of the instructions and their subjective criteria for "best clarity" may have influenced the outcomes. Third, since the study was conducted in a controlled laboratory setting, further research is needed to evaluate the approach's performance in real-world clinical or remote screening environments. It should be noted that the age of participants could influence their ability to perform the DIY approach. For instance, children would probably be unable to follow the procedure independently, and older adults might face physical or cognitive limitations that could affect their performance. In any case, successful DIY refraction would likely require the participant’s cooperation, understanding of the procedure, and sufficient motivation to carry it out effectively.

Recent developments in subjective refraction technology have already introduced hybrid devices that combine automated optimization algorithms with limited clinician supervision.^26^^,^^29^^,^^30^ These systems have shown promising results in maintaining refractive accuracy, reducing chair time and examiner dependency. Integrating such semi-automated elements into patient-driven refraction protocols could represent a natural next step toward scalable, accessible eye-care solutions. Future studies could explore hybrid approaches that combine automated or software-based feedback with clinician supervision to further optimize the balance between accuracy, time efficiency, and accessibility.

In summary, the results suggest that while the optometrist’s guidance introduces subtle differences in the subjective refraction—particularly in the control of accommodation and consistency of timing. These findings support the potential of simplified, patient-driven subjective refraction systems as complementary tools in clinical optometry, especially in scenarios where professional resources are limited or where remote refraction may be beneficial.

## Funding

Ministerio de Universidades FPU20/05624 and EST24/00228.

## Declarations

Nothing to declare.

## Data availability

The data underlying the results presented in the study are available from Zenodo (https://doi.org/10.5281/zenodo.17349090).

## Declaration of competing interest

Authors have no proprietary interest in any of the materials mentioned in this article.
